# Operative Versus Nonoperative Treatment of Z-Type Clavicle Shaft Fractures in Adolescents: A Retrospective Study

**DOI:** 10.3390/children12101278

**Published:** 2025-09-23

**Authors:** Iulia Dobrin, Colin Van Wagoner, Sami Azeroual, Joseph Leider, Ehab Saleh

**Affiliations:** 1 School of Medicine, Oakland University William Beaumont, Rochester, MI 48309, USA; colinvanwagoner@oakland.edu (C.V.W.); sazeroual@oakland.edu (S.A.); 2 Department of Orthopedic Surgery, William Beaumont University Hospital, School of Medicine, Oakland University William Beaumont, Royal Oak, MI 48073, USA; joseph.leider@corewellhealth.org (J.L.); ehab.saleh3@corewellhealth.org (E.S.)

**Keywords:** musculoskeletal disorders, clavicle fractures, fracture management

## Abstract

Background: There are differing opinions in the literature regarding the optimal treatment modality for adolescents with completely displaced, complex clavicle fractures. This study aims to determine outcome differences between surgical and non-surgical treatment for adolescent Z-type clavicle fractures and to ascertain if differences exist in outcomes between the two interventions. Methods: This was a single-center, retrospective chart review performed at a level 1 trauma center. Inclusion criteria included pediatric patients ages 12 to 16 years who presented with a comminuted, displaced clavicle shaft fracture with a comminuted fragment more than 1 cm in length and were treated either operatively or nonoperatively between January 2019 and December 2022. The outcomes were radiographic union status (i.e., union versus non-union versus malunion), follow-up period, shoulder range of motion, return to athletic activities, and patient reported pain level. Results: Of the 24 patients, 11 were treated surgically and 13 non-surgically. Patients who were treated surgically were more likely to be older (mean 1.5 years, *p* = 0.039) and have a longer follow-up by 9 months average duration compared to the cohort treated non-surgically (*p* = 0.0009). There was no significant difference between patient reported pain, radiographic union status, return to athletic activity, or shoulder range of motion between the cohorts. The small sample size and retrospective study design limits the statistical power of our results. Conclusions: The decision between treating these complex fractures operatively versus nonoperatively should be left to a lengthy discussion between the surgeon, parents, and the patient.

## 1. Introduction

Clavicle fractures are common in the adult and pediatric populations [1]. Yet, the literature describes differences regarding treatment in such fractures between these populations, favoring operative treatment for adults and nonoperative treatment for pediatric patients [2]. There is particular uncertainty for treating a subgroup of adolescent clavicle fractures known as the Z-type comminuted fracture. This fracture pattern is a closed, completely displaced and complex fracture pattern with a displaced and vertically rotated butterfly fragment forming a “Z” with the rest of the clavicle [3]. The complexity of this facture lends it to having different healing rates than other midshaft clavicle fractures. This subtype of clavicle fractures is commonly treated surgically due to concerns regarding time to healing and deformity [4]. This stands in contrast to an increasing body of literature indicating equivalent outcomes in treating adolescent clavicle fractures surgically versus nonsurgically [5]. However, there is still some controversy in the literature regarding the treatment of significantly displaced adolescent clavicle fractures [6]. Since adolescents are nearing skeletal maturity with less chances for fracture remodeling, the ideal treatment of displaced fractures for this age group remains controversial [5].

Currently, surgical treatment is primarily indicated for closed, displaced clavicle fractures if there is greater than 2 cm of overlap, superior displacement with skin tenting, associated neurovascular injury, or a floating shoulder [2]. However, there are no randomized controlled trials available to guide the surgical treatment of adolescent clavicle fractures [2]. Research on adult clavicle fractures is not applicable because adolescents rarely have clavicle nonunion or symptomatic malunion, which are the common concerns with nonoperative treatment in adult clavicle fractures [1,7]. There is a limited body of literature exploring this specific subset of clavicle fractures in adolescents, and a larger body of conflicting literature regarding all displaced adolescent clavicle fractures [4,8,9,10]. Sabatini et al. [4] most recently looked at Z-type fractures in patients ages 10–18 years old at eight different pediatric centers and found no benefit from surgery when compared to nonoperative treatment.

The aim of this study is to examine the difference in outcomes between nonoperative and operative treatment in adolescent Z-type clavicle fractures. Outcomes included patient reported level of pain, return to athletic activity, shoulder range of motion, and length of follow-up period.

## 2. Materials and Methods

This study was a single-center, retrospective chart review performed at a level 1 trauma center. Institutional Review Board approval was granted for the completion of this project. The electronic medical record was queried for patient encounters between January 2019 and December 2022. Charts were queried for adolescents with ICD-10-CM diagnosis codes S42.021A and S42.022A for displaced closed fracture of shaft of right and left clavicle, respectively.

Inclusion criteria included: patients 12 to 16 years of age, seen at the center between 1 January 2019 and 31 December 2022, diagnosis of a comminuted displaced clavicle shaft with a butterfly fragment more than 1cm in length, with >35 degrees of angulation relative to the long axis of the clavicle, in-patient admission with surgical treatment, and out-patient cases treated non-surgically. Excluded patients included those younger than 12 years of age and older than 16 years of age, and those with open clavicle fractures.

Data collected from the electronic medical record included patient MRN, age at injury, gender, hand dominance, treatment modality (operative vs. nonoperative), post-operative radiographic evaluation (union, non-union, and malunion), time needed to achieve radiographic union, follow-up period, shoulder range of motion, return to athletic activities, and patient reported level of pain.

Patients were separated into operative and nonoperative groups for comparison. Statistical analysis was conducted to evaluate differences between the collected variables between the two groups. A Wilcoxon rank sum test was used to analyze quantitative values while a Fisher Exact test was conducted to analyze categorical variables. The statistical test utilized for a given variable is denoted by a numerical superscript over the associated *p*-value in the results table.

## 3. Results

**Figure 1:**
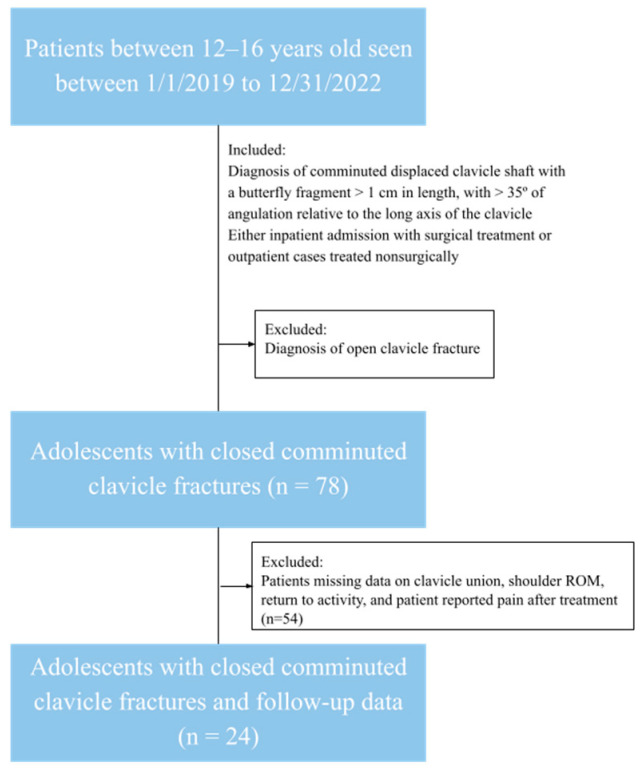
A STROBE diagram of participant selection.

The chart query identified 78 patients who fit the inclusion criteria. A total of 54 additional patients were excluded due to a lack of sufficient data required for statistical comparison, as seen in Figure 1. The remaining cohort was 24 unique patients with no bilateral injuries or fractures. These patients were separated into an operative and nonoperative group, wherein 11 patients underwent surgical treatment and 13 patients received non-surgical treatment. As seen in Table 1, the mean age of the cohort was 14.3 years old, and 20/24 (83.3%) of the total patient population was male. Regarding comparisons between the surgical and non-surgical groups, the surgical group was 1.5 years older on average (*p* = 0.039). The surgical group also had a significantly greater duration of follow-up of about a year compared to three months for the non-surgical group (*p* = 0.0009). There were no significant differences in patient reported pain (*p* = 0.082), patient sex (*p* = 0.60), fracture union vs. non-union (*p* = 1.0000), or return to activity (*p* = 0.46), with all patients within the study cohort reporting a return to full range of motion (ROM). Fracture union means the bone ends join in the correct position, non-union means the fracture fails to heal, and malunion means the bone ends heal in the wrong position.

## 4. Discussion

Our data showed statistically significant differences between surgical and non-surgical cohorts when it comes to age and duration of follow-up. The mean and median age for the surgical cohort were 15.1 and 16 years old, respectively. The mean and median age for the nonsurgical group were 13.6 and 14 years old, respectively. The surgical group being significantly older than the nonsurgical group may be an indicator of surgeon discretion regarding each patients’ bone remodeling potential. The follow up time was on average 9.2 months longer in the surgical cohort than the non-surgical cohort. It is unclear if this is due to patient concern about the healing or a standard of care post-operative follow-up visit depending on treatment modality. There were no significant differences in gender, return to activity, range of motion, patient reported pain, or radiographic union status between the two groups. There were three patients in the surgical group reporting some level of pain, with two patients citing it as minimal pain and one patient as moderate pain. Although there was no statistically significant difference in patient reported pain (*p* = 0.082), the *p*-value is close enough to being significant that warrants mentioning. There is the possibility of clinical significance of this pain, as well as the potential for a type II error. The non-surgical group all stated they had no residual pain. Because of the small sample size and relative clinical insignificance of these differences, this supports current literature that states there are inconsequential differences between treating adolescent Z-type clavicle fractures operatively and nonoperatively in regards to pain and union rate.

### 4.1. Comparison to Literature

Girls achieve 80% of clavicle length by age 9 and boys by age 12, meaning adolescents past these ages with clavicle fractures have limited remodeling potential if a malunion occurs [2]. Because of this and conflicting findings in the literature, adolescent closed clavicle fractures specifically are a gray area when it comes to preferred treatment modalities. Depending on the patient’s age, there may be little to no remodeling potential left. Clinically, these types of fractures are seen in the 14–16 year-old range. Due to the controversy on best indications for surgical intervention for closed clavicle fractures in adolescents, we looked at a specific subset of fractures: the Z-type fracture. A Z-type clavicle fracture is a closed, comminuted fracture with a displaced and vertically rotated butterfly fragment of the clavicle between the major fragments, forming the “Z”-like shape it is named after [3]. Z-type clavicle fractures are often treated surgically in adults due to concerns of gross deformity, delayed union, or nonunion [4].

Some studies conclude that surgical treatment is the best option: Vander Have et al. [10] looked at 42 adolescent patients with closed midshaft clavicle fractures and found symptomatic malunion to occur at higher rates than previous literature had described, occurring in 5 of the 25 patients treated nonoperatively. They concluded that plate fixation “reliably restores length and alignment” in this patient population, has a shorter time to union, and eliminates symptoms associated with malunion, and therefore should be indicated in such fractures [10]. Another study also found good outcomes in operative treatment of displaced midshaft clavicle fractures in pediatric patients, however this study did not compare these results to nonoperative treatments of similar fractures [11]. However, both of these studies were published in 2010–2011. Several subsequent studies have since demonstrated no difference in outcomes in these types of adolescent fractures between nonoperative and operative treatment [1,9,12,13].

Open reduction and internal fixation (ORIF) has been proven to be a safe operation in displaced clavicle shaft fractures in children [14]. However, complications can still arise with both nonoperative and operative modes of treatment, increasing the importance of weighing potential risks against potential benefits of treatment options for each patient. Potential complications of treating clavicle fractures nonoperatively include symptomatic or cosmetic malunion, brachial plexus irritation, or thoracic outlet syndrome in cases of significant shortening from malunion [5]. Potential complications of clavicle fractures being treated operatively include implant-related problems like hardware irritation that may require another surgery for hardware removal, risk of infection, nonunion, numbness in the distribution of the supraclavicular nerve, and refracture [5]. There is a trend of increasing surgical management of closed clavicle fractures in pediatrics and adolescents, despite little support from the literature [6,15]. In an epidemiological study of adolescent clavicle fractures, over half of fractures in the cohort were completely displaced, and nearly one-third of these completely displaced fractures were surgically treated with ORIF [16]. In addition, nearly one-fifth of those clavicle fractures were comminuted, although there was no specification how many of these were Z-type fractures specifically [16]. Furthermore, Yang et al. [17] argues there is a statistically significant increase in surgical treatment amongst adolescents ages 15–19 when compared to younger pediatric and early adolescent patients ages 10–14 [17]. One reason surgery may be preferred by pediatric orthopedic surgeons even in situations where it is not proven by research to have superior outcomes may be based on anecdotal quicker return to activity, lower rates of malunion, and lower patient-reported pain amongst their surgically treated patients, without consideration of the other patient factors differentiating these patients from non-surgically treated patients.

More recently, a study looked at a cohort of 16 adolescent patients who had radiographic malunion after nonoperative treatment of a midshaft clavicle fracture. After controlling for hand dominance, there was a statistically significant reduction in forward flexion and abduction compared to each patient’s uninjured side. Additionally, one of the 16 patients required corrective osteotomy due to symptomatic malunion [7]. Despite these findings, and all of these patients having displacement > 2 cm, which often indicates the need for surgical intervention, the authors concluded there was no clinically meaningful loss of shoulder motion or abduction/adduction strength [7]. A more recent literature review by Cole et al. [6] showed a rising incidence in surgical treatment of closed clavicle fractures in adolescents between 2011–2021. This finding comes in the same ten-year period as several studies that found no differences between nonoperative and operative outcomes, suggesting that the literature and typical clinical practice are not aligned [6].

### 4.2. Clinical Implications

Compared to the existing literature, our study discovered similar findings indicating there is no significant difference between nonoperative and operative outcomes in this specific type of fracture for adolescents. The significant differences we did find were not deemed clinically significant due to the small group of participants and minimal impact on long-term outcomes for these patients. Based on those findings, in our clinical practice, we do have a lengthy discussion with the parents and patient regarding which treatment option to choose for a Z-type clavicle fracture. We do inform them about the similarities of final good outcomes between the operative and nonoperative options. We explain to them that surgery usually results in faster recovery in regards to shoulder range of motion, as well as faster and more predictable healing than nonoperative treatment. Regarding the malunion and gross deformity associated with the nonoperative treatment, we do explain to them that although surgery usually addresses that, the drawback would be the presence of a long scar and the possibility of numbness at the surgical site.

### 4.3. Limitations

Limitations of our study include the retrospective nature of the study and difficulty of controlling for possible confounding variables. In the overall cohort, many patients, most of whom were treated non-surgically, had a shorter follow-up. We found that patients treated surgically were more likely to be older and have a longer follow-up period than patients treated non-surgically. This affects our ability to fairly compare the operative and non-operative groups. Due to the absence of randomization, we cannot establish a causative relationship. The operative cohort was almost two years older than the nonoperative group, which can also affect outcomes due to the operative patients’ growth plates being more closed on average, leading to less remodeling potential compared to the nonoperative cohort. We also had coarse time-to-event resolution that limits our ability to stratify whether the return to activity allowed for full-contact sport or was limited to no-contact activity, which would have been helpful for guiding family counseling in deciding treatment modality. In addition, the cohort size was limited due to 54 of the 78 patients having insufficient data to include in the analysis. While a small cohort, 24 patients is comparable to prior literature on the topic, such at Vanderhave et. al. and Bae et al., who had a cohort of 42 and 16 patients, respectively [7,10].

### 4.4. Future Directions

Further research is warranted to investigate adolescent operative and nonoperative recovery after Z-type clavicle fractures. We propose a longitudinal study may help to stratify outcomes over time, enabling enhanced understanding of what recovery looks like in the short-, medium-, and long-term. Likewise, studies controlling for patient age may better account for the differences in remodeling potential within each adolescent cohort. Studies looking at Quick Disability of Arm, Shoulder, and Hand (QuickDASH) questionnaire scores, which measures physical function and symptoms in patients with upper limb musculoskeletal disorders, or equivalent standardized questionnaires would also provide more comprehensive outcomes for comparison.

### 4.5. Conclusions

In conclusion, our study finds no clear advantage of adolescents being treated surgically versus non-surgically for Z-type clavicle fractures. The results of this study may be used to guide further prospective research and clinician discussions regarding best practices for treating adolescent Z-type clavicle fractures and other types of closed, displaced clavicle fractures amongst adolescent patients. Considering the minimal differences within our study and the continued controversy within the literature regarding surgical and non-surgical treatment of Z-type fractures in adolescents, the decision between treating these complex fractures operatively versus nonoperatively should be left to a lengthy discussion between the surgeon, parents, and the patient.

## Figures and Tables

**Table 1:** A comparison of patients with Z-type clavicle fractures undergoing surgical versus non-surgical treatment with mean (SD) and median (IQR) values provided for continuous data and raw values and percentages provided for categorical data.

Treatment						
	Surgical (N = 11)	Non-Surgical (N = 13)	Total (N = 24)	*p*-Value	95% Confidence Interval	Risk Difference (95% CI)
Age				**0.039** ^1^	(13.628–14.972)	
N	11	13	24			
Mean (SD)	15.1 (1.30)	13.6 (1.71)	14.3 (1.68)			
Median (IQR)	16.0 (14.0, 16.0)	14.0 (12.0, 15.0)	14.5 (12.0, 16.0)			
						
Sex, n (%)				0.60 ^2^		
Female	1/11 (9.1%)	3/13 (23.1%)	4/24 (16.7%)			
Male	10/11 (90.9%)	10/13 (76.9%)	20/24 (83.3%)			
						
Union Status, n (%)				1.00 ^2^		
Union	6/8 (75.0%)	7/7 (100.0%)	13/15 (86.7%)			
Delayed Union	1/8 (12.5%)	0/7 (0.0%)	1/15 (6.7%)			
Non-Union	1/8 (12.5%)	0 (0.0%)	1/15 (6.7%)			
Missing	3	6	9			
						
Shoulder ROM, n (%)						
Full	11/11 (100.0%)	13/13 (100.0%)	24/24 (100.0%)			
						
Return to Activity, n (%)				0.46 ^2^		
Full	10/11 (90.9%)	13/13 (100.0%)	23/24 (95.8%)			3.5 (0.16–78.19)
Partial	1/11 (9.1%)	0/13 (0.0%)	1/24 (4.2%)			
						
Patient Reported Pain, n (%)				0.082 ^2^		
No Residual Pain	8/11 (72.7%)	13/13 (100.0%)	21/24 (87.5%)			8.17 (0.47–142.77)
Minimal Pain	2/11 (18.2%)	0/13 (0.0%)	2/24 (8.3%)			
Moderate Residual Pain	1/11 (9.1%)	0/13 (0.0%)	1/24 (4.2%)			
						
Follow Up (Months)				**0.0009** ^1^	(4.531–10.469)	
N	11	13	24			
Mean (SD)	12.5 (8.29)	3.3 (2.46)	7.5 (7.42)			
Median (IQR)	12.0 (4.0, 22.0)	3.0 (2.0, 3.0)	3.5 (3.0, 11.5)			

^1^ Wilcoxon rank sum *p*-value; ^2^ Fisher Exact *p*-value.

## Data Availability

The data presented in this study are available on request from the corresponding author due to privacy concerns related to medical records.
